# The Effect of Adverse Patient Characteristics on Perioperative Outcomes in Open and Robot-Assisted Radical Prostatectomy

**DOI:** 10.3389/fsurg.2020.584897

**Published:** 2020-11-10

**Authors:** Mike Wenzel, Felix Preisser, Lena H. Theissen, Clara Humke, Maria N. Welte, Clarissa Wittler, Luis A. Kluth, Pierre I. Karakiewicz, Felix K. H. Chun, Philipp Mandel, Andreas Becker

**Affiliations:** ^1^Department of Urology, University Hospital Frankfurt, Frankfurt, Germany; ^2^Cancer Prognostics and Health Outcomes Unit, Division of Urology, University of Montreal Health Center, Montreal, QC, Canada

**Keywords:** prostate cancer, perioperative outcome, BMI, surgical margin, neurovascular bundle preservation, prostate volume, blood loss, OR time

## Abstract

**Objective:** To analyze the effect of adverse preoperative patient and tumor characteristics on perioperative outcomes of open (ORP) and robot-assisted radical prostatectomy (RARP).

**Material and Methods:** We retrospectively analyzed 656 patients who underwent ORP or RARP according to intraoperative blood loss (BL), operation time (OR time), neurovascular bundle preservation (NVBP) and positive surgical margins (PSM). Univariable and multivariable logistic regression models were used to identify risk factors for impaired perioperative outcomes.

**Results:** Of all included 619 patients, median age was 66 years. BMI (<25 vs. 25-30 vs. ≥30) had no influence on blood loss. Prostate size >40cc recorded increased BL compared to prostate size ≤ 40cc in patients undergoing ORP (800 vs. 1200 ml, *p* < 0.001), but not in patients undergoing RARP (300 vs. 300 ml, *p* = 0.2). Similarly, longer OR time was observed for ORP in prostates >40cc, but not for RARP. Overweight (BMI 25-30) and obese ORP patients (BMI ≥30) showed longer OR time compared to normal weight (BMI <25). Only obese patients, who underwent RARP showed longer OR time compared to normal weight. NVBP was less frequent in obese patients, who underwent ORP, relative to normal weight (25.8% vs. 14.0%, *p* < 0.01). BMI did not affect NVPB at RARP. No differences in PSM were recorded according to prostate volume or BMI in ORP or RARP. In multivariable analyses, patient characteristics such as prostate volume and BMI was an independent predictor for prolonged OR time. Moreover, tumor characteristics (stage and grade) predicted worse perioperative outcome.

**Conclusion:** Patients with larger prostates and obese patients undergoing ORP are at risk of higher BL, OR time or non-nervesparing procedure. Conversely, in patients undergoing RARP only obesity is associated with increased OR time. Patients with larger prostates or increased BMI might benefit most from RARP compared to ORP.

## Introduction

Radical prostatectomy (RP) represents one of the treatment standards for localized and locally advanced prostate cancer (1, 2). In addition to local tumor control, preservation of functional anatomic structures as the neurovascular bundle represent key challenges of RP.

It is commonly accepted that adverse preoperative tumor characteristics such as clinical stage (cT), Gleason score and PSA are associated with adverse perioperative results, such as increased blood loss, increased operation (OR) time, positive surgical margins (PSM) or lower probability of neurovascular bundle preservation (NVBP) (3, 4). However, non-tumor-related adverse patient characteristics such as obesity or high prostate volume might also deteriorate perioperative results (5, 6). This seems even more relevant as adverse perioperative results represent strong predictors of long term oncological and functional outcomes after RP (7–10).

Several studies suggest that robotic assisted RP (RARP) might circumvent adverse outcomes like PSM, excessive blood loss, particularly in obese or frail or elderly patients (6, 7, 11, 12).

These considerations are even more important in the context of inverse stage migration and a growing proportion of men with unfavorable patient characteristics—such as obesity—undergoing RP.

Therefore, we investigated predictors of unfavorable perioperative outcomes with a special focus on preoperative adverse tumor and patient related characteristics (i.e., high BMI and high prostate volume) and surgical approach (open RP (ORP) vs. RARP).

## Materials and Methods

### Study Population

After approval of the ethic committee, all 656 patients who underwent ORP or RARP at the Department of Urology at Frankfurt University Hospital between 2014 and 05/2020 were consecutively identified in the institutional database and evaluated retrospectively. Indications for RP was biopsy confirmed prostate cancer. All surgeons, who performed RP in this patient cohort, were experienced surgeons trained in high-volume prostate cancer centers. Exclusion criteria for the analysis was an unknown BMI, prostate volume or pathological surgical margin status (*n* = 37).

### Statistical Analysis

Main objective was to investigate the effect of patient characteristics on perioperative outcome. Descriptive statistics included frequencies and proportions for categorical variables. Means, medians and interquartile ranges (IQR) were reported for continuously coded variables. The Chi-square test was used for statistical significance in proportions' differences. The *t*-test and Kruskal-Wallis test examined the statistical significance of means' and distributions' differences.

To investigate the influence of prostate volume on perioperative outcome, two groups (≤ Median prostate size vs. > Median) were tested. Further, in order to investigate the influence of BMI (in kg/m^2^), the BMI was divided into three categories, namely normal weight (<25), overweight (≥25–30) and obese (≥30).

Increased blood loss (> Median blood loss), increased OR time (>median OR time), non-nerve sparing procedure (NVBP) and PSM were considered as adverse operative outcomes and represented clinical endpoint of this study. NVBP was performed as previously described (3).

Four sets of univariable and multivariable logistic regression models were fitted to test the relationship between preoperative patient characteristics and predict increased perioperative blood loss (defined as > Median blood loss), increased OR time (defined as > Median OR time), and NVBP as well as PSM. Univariable and multivariable models were set for the covariables of age at surgery, BMI, prostate specific antigen (PSA) at diagnosis, prostate volume, cT stage, biopsy Gleason group grade (grouped by 6 vs. 7 vs. 8-10), surgery approach (ORP vs. RARP) and either blood loss, operation time, NVBP, or PSM.

All tests were two sided with a level of significance set at *p* < 0.05 and R software environment for statistical computing and graphics (version 3.4.3) was used for all analyses.

## Results

### Patient Characteristics

In total, 619 consecutive patients were included in our analysis and patient characteristics stratified by prostate volume and BMI are summarized in Tables 1, 2. Median age was 66 years and median prostate volume was 40cc (≤ 40: 30cc vs. >40: 56cc). Moreover, the median BMI was 26.3 kg/m^2^, with 23.6 kg/m^2^, 26.9 kg/m^2^ and 32.0 kg/m^2^ in the normal weight, overweight and obese group, respectively. Median PSA was 7.7 ng/ml. ORP was performed in 36.8% (*n* = 228) and RARP in 63.2% (*n* = 391) patients.

**Table 1:** Patient characteristics stratified by prostate volume.

		**Overall *N* = 619**	**Prostate volume ≤40cc *N* = 367 (58.2%)**	**Prostate volume >40cc *N* = 267 (41.8%)**	***P* value**
Age (years)	Median (IQR)	66 (60–71)	66 (60–70)	67 (62–72)	<0.01
BMI (kg/m^2^)	Median (IQR)	26.3 (24.3–29.1)	26.2 (24.3–28.8)	26.5 (24.2–29.5)	0.6
Prostate volume (ccm)	Median (IQR)	40 (30–52)	30 (25–36)	56 (49–70)	<0.001
iPSA (ng/ml)	Median (IQR)	7.7 (5.6–11.9)	7.2 (5.4–10.9)	8.6 (6.1–13.3)	<0.001
OR time (min)	Median (IQR)	233 (190–272)	227 (188–264)	240 (197–283)	<0.01
OR time ORP (min)	Median (IQR)	226 (193–258)	215 (189–248)	239 (202–274)	<0.01
OR time RARP (min)	Median (IQR)	240 (189–280)	239 (187–276)	244 (197–285)	0.2
Blood loss (ml)	Median (IQR)	400 (300–800)	300 (200–800)	500 (300–1200)	<0.001
Blood loss ORP (ml)	Median (IQR)	1000 (800–1500)	800 (688–1200)	1200 (800–1775)	<0.001
Blood loss RARP (ml)	Median (IQR)	300 (200–300)	300 (200–300)	300 (200–300)	0.2
Gleason grade	6	124 (20.0)	70 (19.4)	54 (20.8)	0.8
	7	355 (57.4)	210 (58.3)	145 (56.0)	
	8–10	140 (22.6)	80 (22.2)	60 (23.2)	
cT stage	cT1c	295 (47.7)	178 (49.4)	117 (45.2)	0.6
	Any cT2	285 (46.0)	162 (45.0)	123 (47.5)	
	≥cT3	30 (4.8)	16 (4.4)	14 (5.4)	
D‘Amico Classification	Low risk	74 (12.0)	45 (12.5)	29 (11.2)	0.9
	Intermediate risk	337 (54.4)	197 (54.7)	140 (54.1)	
	High risk	203 (32.8)	116 (32.2)	87 (33.6)	
Surgical approach	ORP	228 (36.8)	123 (34.2)	105 (40.5)	0.1
	RARP	391 (63.2)	237 (65.8)	154 (59.5)	
Nerve–sparing	Bilateral	406 (65.6)	232 (64.4)	174 (67.2)	0.4
	Unilateral	94 (15.2)	52 (14.4)	42 (16.2)	
	No	97 (15.7)	62 (17.2)	35 (13.5)	
Nerve–sparing ORP	Bilateral	141 (65.6)	75 (65.2)	66 (66.0)	1
	Unilateral	35 (18.1)	19 (16.5)	16 (16.0)	
	No	39 (16.3)	21 (18.3)	18 (18.0)	
Nerve–sparing RARP	Bilateral	265 (69.4)	157 (68.0)	108 (71.5)	0.2
	Unilateral	59 (15.4)	33 (14.3)	26 (17.2)	
	No	58 (15.2)	41 (17.7)	17 (11.3)	
Surgical margin	Negative	447 (72.2)	259 (71.9)	188 (72.6)	0.9
	Positive	172 (27.8)	101 (28.1)	71 (27.4)	
Surgical margin ORP	Negative	159 (69.7)	87 (70.7)	72 (68.6)	0.8
	Positive	69 (30.3)	36 (29.3)	33 (31.4)	
Surgical margin RARP	Negative	288 (73.7)	172 (72.6)	116 (75.3)	0.6
	Positive	103 (26.3)	65 (27.4)	38 (24.7)	

*Descriptive characteristics of 619 patients undergoing radical prostatectomy and stratified by prostate volume. BMI, Body mass index; iPSA, initial Prostate Specific Antigen; ORP, Open Radical Prostatectomy; RARP, Robot–assisted Radical Prostatectomy*.

**Table 2:** Patient characteristics stratified by BMI.

**Varname**		**Normal weight (A) *N* = 217 (35.1%)**	**Overweight (B) *N* = 273 (44.1%)**	**Obese (C) *N* = 129 (20.8%)**	***P* value A vs. B**	***P* value A vs. C**
Age (years)	Median (IQR)	68 (63–72)	66 (59–72)	64 (59–69)	<0.01	<0.001
BMI (kg/m^2^)	Median (IQR)	23.6 (22.3–24.4)	26.9 (25.9–28.4)	32.0 (30.7–34.3)	<0.001	<0.001
Prostate volume (ccm)	Median (IQR)	40 (30–55)	37 (30–50)	40 (30–54)	0.5	0.5
iPSA (ng/ml)	Median (IQR)	8.0 (6.1–11.9)	7.0 (5.4–11.2)	8.1 (5.9–15.2)	0.037	0.6
OR time (min)	Median (IQR)	222 (187–262)	232 (190–270)	251 (204–289)	0.16	<0.001
OR time ORP (min)	Median (IQR)	213 (185–246)	233 (198–260)	238 (206–281)	0.02	<0.01
OR time RARP (min)	Median (IQR)	234 (188–277)	231 (189–275)	256 (198–299)	1	0.02
Blood loss (ml)	Median (IQR)	400 (300–800)	400 (300–800)	400 (300–1000)	0.9	0.6
Blood loss ORP (ml)	Median (IQR)	800 (800–1500)	1000 (800–1500)	1200 (800–1500)	0.8	0.2
Blood loss RARP (ml)	Median (IQR)	300 (200–300)	300 (200–300)	300 (200–350)	0.3	0.5
Gleason grade	6	47 (21.7)	47 (17.2)	30 (23.3)	0.3	0.2
	7	127 (58.5)	165 (60.4)	63 (48.8)		
	8–10	43 (19.8)	61 (22.3)	36 (27.9)		
cT stage	cT1c	100 (46.1)	126 (46.2)	69 (53.5)	0.4	0.3
	Any cT2	102 (47)	134 (49.1)	49 (38.0)		
	≥cT3	13 (6.0)	9 (3.3)	8 (6.2)		
D‘Amico Classification	Low risk	28 (12.9)	26 (9.5)	20 (15.5)	0.5	0.2
	Intermediate risk	122 (56.2)	155 (56.8)	60 (46.5)		
	High risk	65 (30.0)	89 (32.6)	49 (38.0)		
Surgical approach	ORP	82 (37.8)	103 (37.7)	43 (33.3)	1	0.5
	RARP	135 (62.2)	170 (62.3)	86 (66.7)		
Nerve–sparing	Bilateral	146 (67.3)	180 (65.9)	80 (62.0)	0.6	0.5
	Unilateral	28 (12.9)	45 (16.5)	21 (16.3)		
	No	34 (15.7)	40 (14.7)	23 (17.8)		
Nerve–sparing ORP	Bilateral	56 (72.7)	67 (69.1)	18 (43.9)	1	<0.01
	Unilateral	9 (11.7)	19 (19.6)	7 (17.1)		
	No	12 (15.6)	11 (11.3)	16 (39.0)		
Nerve–sparing RARP	Bilateral	90 (68.7)	113 (67.3)	62 (74.7)	1	0.2
	Unilateral	19 (14.5)	26 (15.5)	14 (16.9)		
	No	22 (16.8)	29 (17.3)	7 (8.4)		
Surgical margin	Negative	159 (73.3)	203 (74.4)	85 (65.9)	0.9	0.2
	Positive	58 (26.7)	70 (25.6)	44 (34.1)		
Surgical margin ORP	Negative	58 (70.7)	74 (71.8)	27 (62.8)	1	0.5
	Positive	24 (29.3)	29 (28.2)	16 (37.2)		
Surgical margin RARP	Negative	101 (74.8)	129 (75.9)	58 (67.4)	0.9	0.3
	Positive	34 (25.2)	41 (24.1)	28 (32.6)		

Descriptive characteristics of 619 patients undergoing radical prostatectomy and stratified by weight: Normal weight (BMI <25) vs. overweight (BMI 25–30) vs. obese (BMI ≥ 30).

*BMI, Body mass index; iPSA, initial Prostate Specific Antigen; ORP, Open Radical Prostatectomy; RARP, Robot–assisted Radical Prostatectomy*.

### Perioperative Outcomes

#### Blood Loss

Median blood loss was 400 ml (IQR 300–800). Stratified by prostate volume, there was a significant higher blood loss in prostate volume >40cc vs. ≤ 40cc (500 vs. 300 ml, *p* < 0.001) performing RP in general. This significant difference was also obvious in ORP (1200 vs. 800 ml, *p* < 0.001), whereas no difference was seen in patients undergoing RARP (300 vs. 300 ml, *p* = 0.2). No significant differences were observed in blood loss regarding to BMI groups. Stratified by age categories (> vs. ≤ Median), no significant differences were seen (data not shown). Univariate analyses (Table 3) revealed significant risk factors for increased blood loss for age (Odds ratio (OR): 1.03), prostate volume (OR: 1.02), PSA (OR: 1.03), ≥cT3 (OR: 4.46), Gleason grade 8-10 (OR: 8.46), and no NVBP (OR: 2.71), whereas performing RARP was a protective factor for increased blood loss (OR: 0.01, all *p* < 0.05). After multivariable analyses and adjustment for patient and tumor characteristics, biopsy Gleason 8-10 (OR: 3.40) was an independent predictor of increased blood loss, whereas RARP had a protective effect (OR: 0.01, all *p* < 0.05).

**Table 3:** Univariable and multivariable logistic regression models predicting blood loss > 400 ml (Median).

	**Univariable**			**Multivariable**		
	**OR**	**CI 2.5–97.5%**	***P* value**	**OR**	**CI 2.5–97.5%**	***P* value**
Age	1.03	1.01–1.06	0.03	0.99	0.94–1.04	0.7
BMI	1.01	0.97–1.06	0.6	1.03	0.94–1.12	0.5
iPSA	1.03	1.01–1.04	<0.001	1.00	0.98–1.01	0.5
Prostate volume	1.02	1.01–1.03	<0.001	1.01	1.00–1.03	0.1
cT1c (Ref.)	1	–	–	1 (Ref.)	–	–
cT2	1.44	0.98–2.10	0.06	0.88	0.43–1.76	0.7
≥ cT3	4.46	1.81–12.61	<0.01	0.54	0.11–3.11	0.5
Gleason 6	1 (Ref.)	–	–	1 (Ref.)	–	–
7	1.49	0.90–2.51	0.1	0.99	0.43–2.38	1
8–10	8.42	4.47–16.40	<0.001	3.40	1.12–10.48	<0.01
Surgical approach ORP	1 (Ref.)	–	–	1 (Ref.)	–	–
RARP	0.01	0.01–0.02	<0.001	0.01	0.01–0.02	<0.01
Nerve–sparing bilateral	1 (Ref.)	–	–	1 (Ref.)	–	–
Unilateral	1.27	0.79–2.06	0.3	1.90	0.84–4.27	0.1
No Nerve–sparing	2.71	1.46–5.22	<0.01	0.86	0.22–3.24	0.8

*BMI, Body mass index; iPSA, initial Prostate Specific Antigen; ORP, Open Radical Prostatectomy; RARP, Robot–assisted Radical Prostatectomy; OR, Odds ratio and CI, Confidence interval*.

#### OR Time

Median OR time was 233 min (IQR 190-272). Our analyses recorded a significantly longer OR time in RP for prostates > 40 cc comparing to ≤ 40cc (240 min vs. 227 min, *p* < 0.01). This difference was also seen for ORP (239 min vs. 215 min, *p* < 0.001), whereas RARP showed no significant differences in OR time (244 min vs. 239 min, *p* = 0.2). According to BMI, a longer OR time was observed between normal weighted and obese patients (222 vs. 251 min, *p* < 0.001). Stratified by approach, ORP showed significant longer OR times in the comparison of normal weight vs. overweight and vs. obese patients, whereas in patients undergoing RARP only a difference between normal weight and obese patients was recorded (234 vs. 256 min, *p* = 0.02). In univariable analyses (Table 4), prostate volume, BMI were risk factors for increased OR time (all *p* < 0.05). In multivariable analyses after adjustment for patient and tumor characteristics, prostate volume and BMI were independent predictors of longer OR time (all *p* < 0.05).

**Table 4:** Univariable and multivariable logistic regression models predicting OR time > 233 min (Median).

	**Univariable**			**Multivariable**		
	**OR**	**CI 2.5–97.5%**	***P* value**	**OR**	**CI 2.5–97.5%**	***P* value**
Age	1.00	0.98–1.02	1	1.00	0.98–1.02	0.9
BMI	1.06	1.02–1.11	<0.01	1.06	1.01–1.11	0.01
iPSA	0.99	0.99–1.00	0.2	0.99	0.98–1.00	0.1
Prostate volume	1.01	1.01–1.02	<0.001	1.01	1.00–1.02	<0.01
cT1c (Ref.)	1 (Ref.)	–	–	1 (Ref.)	–	–
cT2	0.66	0.47–0.90	0.01	0.66	0.48–0.94	0.02
≥ cT3	0.71	0.34–1.47	0.4	0.64	0.27–1.52	0.3
Gleason 6	1 (Ref.)	–	–	1 (Ref.)	–	–
7	0.80	0.53–1.21	0.3	0.82	0.53–1.27	0.4
8–10	0.74	0.46–1.19	0.2	0.70	0.39–1.26	0.2
Surgical approach ORP	1 (Ref.)	–	–	1 (Ref.)	–	–
RARP	1.26	0.92–1.74	0.2	1.06	0.72–1.55	0.8
Nerve–sparing bilateral	1 (Ref.)	–	–	1 (Ref.)	–	–
Unilateral	1.71	1.09–2.70	0.02	1.96	1.22–3.20	<0.01
No Nerve–sparing	1.12	0.73–1.73	0.6	1.58	0.94–2.68	0.08

*BMI, Body mass index; iPSA, initial Prostate Specific Antigen; ORP, Open Radical Prostatectomy; RARP, Robot–assisted Radical Prostatectomy; OR, Odds ratio and CI, Confidence interval*.

#### Nerve-Sparing

Overall, bilateral, unilateral, no NVBP and unknown NVBP status was recorded in 65.6% (*n* = 406), 15.2% (*n* = 94), 15.7% (*n* = 97), and 3.6% (*n* = 22), respectively. No significant differences in NVBP were seen across prostate volume strata, neither after stratification into ORP or RARP. NVBP could be performed less frequently in ORP in obese patients compared to normal weighted patients (25.8% vs. 14.0%, *p* < 0.01). No statistically significant differences according to BMI were seen for NVBP in RARP. In univariable analyses (Table 5), PSA (OR: 1.05), cT2 (OR: 2.97), ≥cT3 (OR:12.84), as well as Gleason Grade ≥8 (OR: 5.53, all *p* < 0.05) were found to be a risk factor for a unilateral or no NVBP. In multivariable analyses after adjustment for patient and tumor characteristics, patient age (OR: 1.09), PSA (OR: 1.03), cT2 (OR: 2.67), and cT3 stage (OR: 8.81), Gleason 8-10 (OR: 5.36) were independent predictors unilateral or no NVPB.

**Table 5:** Univariable and multivariable logistic regression model predicting unilateral or no Neurovascular Bundle Preservation (NVPB).

	**Univariable**			**Multivariable**		
	**OR**	**CI 2.5–97.5%**	***P* value**	**OR**	**CI 2.5–97.5%**	***P* value**
Age	1.02	0.99–1.05	0.1	1.09	1.04–1.16	<0.01
BMI	1.04	0.98–1.09	0.2	1.09	0.99–1.20	0.06
iPSA	1.05	1.03–1.65	<0.001	1.03	1.01–1.05	<0.01
Prostate volume	1.0004	0.99–1.01	1	0.99	0.97–1.01	0.2
Blood loss	1.0003	1.00–1.00	0.1	1.00	1.00–1.00	0.5
cT1c (Ref.)	1 (Ref.)	–	–	1 (Ref.)	–	–
cT2	2.97	1.81–5.01	<0.001	2.67	1.13–6.83	0.03
≥ cT3	12.84	5.74–29.3	<0.001	8.81	2.47–32.50	<0.01
Gleason 6	1 (Ref.)	–	–	1 (Ref.)	–	–
7	1.46	0.75–3.06	0.3	1.47	0.38–9.68	0.6
8–10	5.53	2.81–11.77	<0.001	5.36	1.31–36.51	0.04
Surgical approach ORP	1 (Ref.)	–	–	1 (Ref.)	–	–
RARP	0.77	0.50–1.19	0.2	0.82	0.30–2.31	0.7

*BMI, Body mass index; iPSA, initial Prostate Specific Antigen; ORP, Open Radical Prostatectomy; RARP, Robot–assisted Radical Prostatectomy; OR, Odds ratio and CI, Confidence interval*.

#### Surgical Margin

PSM were recorded in 27.8% (*n* = 183) patients. No significant differences were found across different prostate volume or BMI strata. In univariable analyses (Table 6), PSA (OR 1.02, CI: 1.01-1.03), cT stage (T2: OR: 1.52; ≥T3: OR: 10.77), as well as Gleason Score ≥8 (OR: 3.46, all *p* < 0.05) were found to be significant risk factors for PSM. In multivariable analyses, cT3 stage (OR: 1.89), PSA (OR: 1.02) and Gleason Score ≥8 (OR: 2.21) remained as independent predictors for PSM. Conversely, age was not a significant predictor for PSM.

**Table 6:** Univariable and multivariable logistic regression model predicting Positive Surgical Margin (PSM).

	**Univariable**			**Multivariable**		
	**OR**	**CI 2.5–97.5%**	***P* value**	**OR**	**CI 2.5–97.5%**	***P* value**
Age	1.01	0.98–1.03	0.5	1.02	0.99–1.05	0.3
BMI	1.05	1.00–1.09	0.03	1.03	0–971.08	0.4
iPSA	1.02	1.01–1.03	<0.001	1.02	1.00–1.03	0.02
Prostate volume	1.00	0.99–1.02	0.5	0.99	0.98–1.00	0.3
Blood loss	1.00	1.00–1.00	0.8	1.00	1.00–1.00	0.9
cT1c (Ref.)	1 (Ref.)	–	–	1 (Ref.)	–	–
cT2	1.52	1.05–2.20	0.03	1.23	0.78–1.97	0.4
≥ cT3	10.77	4.81–26.64	<0.001	7.03	2.40–23.75	<0.01
Gleason 6	1 (Ref.)	–	–	1 (Ref.)	–	–
7	1.44	0.88–2.43	0.2	1.05	0.58–1.96	0.9
8–10	3.46	2.02–6.09	<0.01	2.21	1.05–4.76	0.04
Surgical approach ORP	1 (Ref.)	–	–	1 (Ref.)	–	–
RARP	0.74	0.52–1.05	0.1	1.72	0.93–3.25	0.1

*BMI, Body mass index; iPSA, initial Prostate Specific Antigen; ORP, Open Radical Prostatectomy; RARP, Robot–assisted Radical Prostatectomy; OR, Odds ratio and CI, Confidence interval*.

## Discussion

Radical prostatectomy currently remains the gold standard in the treatment of localized and resectable locally-advanced prostate cancer (1, 2). Local tumor control with negative surgical margins as well as the preservation of functionality represent the main goals of RP. We hypothesized that -beside the known effect of preoperative tumor characteristics- adverse non-tumor-related patient characteristics (i.e. high BMI and high prostate volume) deteriorate perioperative outcomes of RP. To test this hypothesis, we examined all 656 patients undergoing RP at our institution since 2014 and investigated several noteworthy findings.

First, our data show important findings according to blood loss. Here, patients with bigger prostates are at risk for increased blood loss. Interestingly, this holds true only for an open approach (ORP), while patients with bigger prostates undergoing RARP are not at higher risk for increased blood loss. While several studies showed decreased blood loss in RARP compared to ORP (13, 14), our study is the first to demonstrate a clear benefit regarding blood loss in patients with big prostates. In contrast to previous studies, our data did not suggest a correlation of BMI and blood loss (15). Potential reason for this discrepancy may stem from different stratifications according to blood loss and weight were used (6, 16). For example, Murakami et al. defined overweight at a BMI ≥ 25 kg/m^2^ (16). As shown in previous studies, tumor characteristics such as cT-stage (cT1c vs. ≥cT3) and Gleason Grade ≥8 were confirmed as independent predictors for increased blood loss, which can be explained by an increased complexity of the surgery (17). These findings are even more meaningful regarding to the fact that high intraoperative blood loss might be associated with a worse outcome of postoperative erectile and continence function (4).

Second, important results could be recorded according to OR time. Patients with large prostates had prolonged OR time. Interestingly, as already seen for the perioperative blood loss, this holds true for ORP but not for RARP. Similarly, BMI was an independent predictor for prolonged OR time Moreover, our data suggest that RARP may compensate prolonged OR time for normal weight vs. overweight patients, whereas OR time significantly increases in obese patients. In general, our data are congruent to the results of Mandel et al., who were able to show prolonged OR time for overweight and obese patients undergoing ORP compared to normal weighted patients, using the same BMI stratification (6). Additionally, comparing our RARP data, Murakami et al. showed differences in RARP operation time for BMI >25 kg/m^2^, but the authors stratified patients to BMI <25 kg/m^2^ vs. ≥25 kg/m^2^ only. Thus, patients with BMI >30 kg/m^2^ might have strongly affected differences in this cohort (16).

Third, according to NVBP, significant differences in our cohort could be recorded between normal weighted and obese patients undergoing ORP. Specifically, obese patients undergoing ORP were at higher risk to undergo a non-nerve sparing procedure, while this was not observed in RARP patients. This may be explained by the better visual conditions of RARP in obese patients, as already reported by Beyer et al. (18). In multivariable adjusted analyses higher BMI failed (*p* = 0.06) to be an independent predictor of none or unilateral NVBP and only age could be investigated as an independent predictor. Intuitively, our data confirm previous studies, showing that adverse tumor characteristics reduce the chance of NVBP (19–21).

Fourth, non-regarding of the surgical approach (ORP vs. RARP) prostate volume and BMI did not represent risk factors for PSM in patients undergoing RP. However, there is some evidence in literature that there may be a difference in PSM in larger prostates and especially in ORP (22, 23). These differences are mainly driven by very large prostate volumes and low patient numbers within those groups. According to different BMI categories, rates of PSM did not reach statistical significance with respect to our data. This could be explained by limited sample size and that the obese subgroup had the fewest number of patients. However, other studies suggest that there might be a detrimental effect of high BMI on PSM (5, 6, 24).

Fifth, after comparing different age groups, our data demonstrate that age has no detrimental effect on most perioperative outcomes. Nonetheless, age was an independent predictor of for less bilateral NVPB. While some studies provide differences in age categories within patients undergoing RP, these are mainly studies comparing very young patients or elderly cohorts and do not reflect the median patient age of prostate cancer diagnosis (25, 26).

Our study has several limitations. First, it is based on retrospective analyses. Second, the selection of the surgical approach (ORP or RARP) probably already represents a selection bias regarding to certain patient and tumor characteristics. Third, both surgical approaches represent methods with different learning curves and surgery was performed by different surgeons. The data described here does not provide information on the surgical volume. Moreover, no information about prostate configuration were available and about the location of PSM. NVPB was offered to every patient including frozen section since 11/2017. Before 2017 selection for NVBP was based on preoperative and intraoperative findings such as digital rectal examination, number and location of positive biopsies, preoperative PSA and if available MRT findings. Furthermore, results of high-volume centers are not necessarily applicable to low-volume care provider, which might limit the generalizability of our findings (27). Finally, our data do not provide data about patient's comorbidities. However, due to the primary selection of patients for RP instead of surveillance or radiation therapy we suggest that all patients had a favorable heath status with a life expectancy of more than 10 years.

Taken together, we can conclude that adverse patient and tumor characteristics have a significant impact on perioperative outcome. In particular, patients with larger prostates and obese patients undergoing ORP are at risk for adverse clinical outcomes like increased BL, OR time or non-nervesparing procedure. Conversely, in patients undergoing RARP, only obesity is associated with increased OR time. Therefore, patients with larger prostates or increased BMI might benefit most from RARP compared to ORP. Finally, BMI and prostate volume are independent predictors for worse perioperative outcomes such as prolonged OR time.

## Data Availability Statement

The raw data supporting the conclusions of this article will be made available by the authors, without undue reservation.

## Ethics Statement

The studies involving human participants were reviewed and approved by Ethikkommission Universitätsklinikum Frankfurt. Written informed consent for participation was not required for this study in accordance with the national legislation and the institutional requirements.

## Author Contributions

MW: manuscript writing/editing, protocol/project development, data collection or management, data analysis. FP: data analysis, protocol/project development. LT and CH: data collection or management. MNW: manuscript writing/editing, data collection or management. CW: data analysis, data collection or management. LK: manuscript writing/editing. PK and FC: manuscript writing/editing, protocol/project development. PM and AB: manuscript writing/editing, protocol/project development, data analysis. All authors contributed to the article and approved the submitted version.

## Conflict of Interest

The authors declare that the research was conducted in the absence of any commercial or financial relationships that could be construed as a potential conflict of interest.
